# From Hypercoagulability to Hypocoagulability: Dynamic ROTEM Phenotypes and Outcomes in Sepsis and Septic Shock

**DOI:** 10.3390/jcm15208089

**Published:** 2026-10-18

**Authors:** Matteo Guarino, Francesco Luppi, Roberto De Giorgio, Michele Domenico Spampinato

**Affiliations:** 1 Department of Translational Medicine, University of Ferrara, 44124 Ferrara, Italy; grnmtt@unife.it (M.G.); francesco.luppi@unife.it (F.L.); spmmhl@unife.it (M.D.S.); 2 Emergency Department, St. Anna University-Hospital of Ferrara, 44124 Ferrara, Italy

**Keywords:** haemostatic phenotypes, rotational thromboelastometry, sepsis, sepsis-induced coagulopathy, septic shock, viscoelastic testing

## Abstract

**Background/Objectives**: Sepsis is characterized by profound and dynamic alterations of haemostasis, ranging from hypercoagulability and fibrinolysis shutdown to mixed patterns, hypocoagulability, and overt disseminated intravascular coagulation. Conventional coagulation tests provide only a partial representation of these changes. Rotational thromboelastometry (ROTEM) offers a whole-blood, dynamic assessment of clot initiation, propagation, firmness, and lysis, potentially allowing for more accurate haemostatic phenotyping. **Methods**: A narrative review of the literature was performed using PubMed, Scopus, and EMBASE from database inception to August 2026. Studies addressing sepsis or septic shock and rotational thromboelastometry, thromboelastometry, viscoelastic testing, coagulation phenotypes, fibrinolysis, disseminated intravascular coagulation, and clinical outcomes were considered. Additional references were identified through manual screening of relevant articles and reviews. **Results**: The available evidence indicates that ROTEM can identify distinct haemostatic phenotypes in sepsis, including hypercoagulability, fibrinolysis shutdown, mixed hypo-hypercoagulable patterns, and hypocoagulability. Hypercoagulable profiles are frequently reported in early or less advanced sepsis, whereas hypocoagulability and reduced clot firmness are increasingly observed in septic shock and advanced coagulopathy. Impaired fibrinolysis and hypocoagulable profiles have been associated with greater organ dysfunction and mortality. Importantly, discordance between conventional coagulation tests and ROTEM is common, supporting the value of integrated whole-blood assessment. However, most studies are observational, heterogeneous, and based on single time-point measurements. **Conclusions**: ROTEM should be regarded primarily as a complementary tool for dynamic haemostatic phenotyping and risk stratification in sepsis and septic shock. Future prospective studies should determine whether serial ROTEM trajectories provide incremental prognostic value and can identify subgroups potentially amenable to phenotype-guided therapeutic strategies.

## 1. Introduction

Sepsis is a life-threatening syndrome resulting from an altered host response to infection leading to acute organ dysfunction [1]. Despite major advances in its recognition and management, sepsis and septic shock remain among the most challenging clinical conditions because of their heterogeneity, rapid evolution, and substantial risk of early deterioration. These issues are particularly relevant in the Emergency Department (ED), where prompt recognition, timely antimicrobial treatment, hemodynamic stabilization, and repeated reassessment are essential [2]. Contemporary international guidelines continue to emphasize early diagnosis and individualized, dynamic management as key components of sepsis care [3].

Among the multiple pathophysiological alterations occurring during sepsis, disruption of haemostasis plays a central role. Infection-driven activation of coagulation, endothelial dysfunction, platelet activation, impairment of natural anticoagulant pathways, and suppression of fibrinolysis generate a complex and evolving haemostatic response [4,5,6,7,8]. Importantly, sepsis-induced coagulopathy should not be interpreted as a uniform bleeding disorder. Septic patients may instead exhibit hypercoagulable, hypofibrinolytic, mixed, or hypocoagulable phenotypes, with progression toward overt disseminated intravascular coagulation in the most severe cases [9,10,11,12].

This haemostatic heterogeneity is only partially captured by conventional coagulation tests, which provide valuable but fragmented information and do not directly assess whole-blood clot development, clot firmness, or fibrinolytic activity [5,13,14]. Rotational thromboelastometry (ROTEM), by dynamically assessing clot initiation, propagation, strength, and lysis, may therefore reveal abnormalities that remain poorly characterized by conventional testing, including hypercoagulability, mixed coagulation patterns, and fibrinolytic shutdown [9,11,12].

The potential role of thromboelastometry in sepsis-induced coagulopathy has recently been comprehensively reviewed [15]. However, an important question remains insufficiently addressed, i.e., whether distinct ROTEM-defined haemostatic phenotypes (particularly their evolution across the course of sepsis) carry different prognostic implications [16,17,18]. The available studies suggest that hypercoagulability, impaired fibrinolysis, mixed patterns, and hypocoagulability may represent clinically distinct states, with the latter phenotypes increasingly associated with organ dysfunction and mortality [11,12,19,20,21,22]. Accordingly, this narrative review focuses specifically on dynamic ROTEM phenotyping in sepsis and septic shock, its relationship with disease severity and clinical outcomes, and the potential prognostic value of moving beyond isolated coagulation measurements toward serial haemostatic assessment.

## 2. Search Strategy

A literature search was conducted in PubMed, Scopus, and EMBASE from database inception to August 2026. The search strategy combined terms related to sepsis and septic shock with viscoelastic coagulation testing, including “sepsis” OR “septic shock” AND “rotational thromboelastometry” OR “ROTEM” OR “thromboelastometry” OR “viscoelastic testing” OR “thromboelastography”. Attention was given to studies evaluating haemostatic phenotypes, coagulation abnormalities, fibrinolysis, disseminated intravascular coagulation, disease severity, and clinical outcomes in adult septic patients. Relevant systematic reviews, meta-analyses, prospective and retrospective observational studies, and selected narrative reviews were considered. Additional references were identified through manual screening of the reference lists of included articles and pertinent reviews. Studies using other viscoelastic haemostatic assays, particularly thromboelastography (TEG), were also considered when they provided relevant pathophysiological or prognostic information. However, ROTEM-specific evidence was preferentially used for assay interpretation and haemostatic phenotype characterization, in keeping with the specific focus of this review.

As this work was conceived as a narrative rather than a systematic or scoping review, no formal eligibility-driven screening process, quantitative study accounting, or PRISMA-style study selection flowchart was planned. The search strategy was intended to provide a broad and clinically oriented synthesis of the available literature rather than an exhaustive systematic inventory of all eligible studies.

Given the narrative design of this review, study selection was based on relevance to the predefined clinical and pathophysiological topics rather than on a formal systematic screening process.

## 3. Pathophysiological Basis of Haemostatic Alterations in Sepsis

Sepsis profoundly disrupts the physiological balance between coagulation, anticoagulant pathways, platelet activity, and fibrinolysis. Rather than representing a purely haemorrhagic disorder, sepsis-induced coagulopathy reflects a dynamic interaction between inflammation and haemostasis, in which initially adaptive mechanisms may progressively become maladaptive and contribute to microvascular thrombosis, organ dysfunction, and, in advanced stages, consumptive coagulopathy and disseminated intravascular coagulation (DIC) [9,23,24,25,26,27,28,29].

### 3.1. Immunothrombosis and Coagulation Activation

Immunothrombosis represents an important component of the innate host response, promoting local containment of pathogens through coordinated activation of leukocytes, platelets, coagulation factors, and the endothelium. In sepsis, however, excessive or sustained activation may shift this protective response toward widespread thrombin generation and microvascular fibrin deposition [30,31,32]. Tissue factor expression, platelet–leukocyte interactions, and neutrophil extracellular trap formation contribute to this procoagulant state, while NETosis and other forms of inflammation-related cellular injury further amplify coagulation activation [23,33,34]. This mechanism provides a pathophysiological basis for the hypercoagulable profiles observed in some patients during early sepsis [10].

### 3.2. Endothelial Dysfunction and Loss of Anticoagulant Balance

The endothelium plays a central role in maintaining haemostatic equilibrium. During sepsis, endothelial activation and glycocalyx disruption favour leukocyte adhesion, tissue factor exposure, and loss of endogenous anticoagulant mechanisms, including the protein C and antithrombin systems. The resulting imbalance promotes thrombin generation and microvascular clot formation, further impairing tissue perfusion and contributing to organ dysfunction [23,27,35,36].

### 3.3. Platelet Activation, Dysfunction, and Consumption

Platelets participate actively in immunothrombosis through adhesion, aggregation, and interaction with neutrophils and endothelial cells. As sepsis progresses, however, persistent activation may coexist with thrombocytopenia, functional impairment, and consumption. Consequently, platelet contribution to clot strength may vary substantially across the septic spectrum, contributing to the coexistence of hypercoagulable, mixed, and hypocoagulable phenotypes [11,12,37,38,39,40].

### 3.4. Fibrinolytic Suppression and Progression Toward SIC and DIC

Suppression of fibrinolysis is another hallmark of septic haemostatic dysfunction. Increased antifibrinolytic activity may lead to persistent clot stability and fibrinolysis shutdown, a pattern associated with greater organ dysfunction and adverse outcomes [22,41]. With increasing disease severity, ongoing coagulation activation, platelet and factor consumption, and endothelial injury may eventually overcome the initial procoagulant response, leading to reduced clot formation and clot strength and, in the most severe cases, overt DIC [26,42,43]. This dynamic evolution provides the biological rationale for interpreting septic coagulopathy through haemostatic phenotypes rather than through isolated laboratory abnormalities.

## 4. Understanding ROTEM in Sepsis: Assays and Key Parameters

Viscoelastic haemostatic assays (VHAs) comprise several whole-blood technologies, most prominently TEG and ROTEM. Both provide dynamic information on clot initiation, propagation, strength, and lysis, but differ in instrumentation, activation systems, assay architecture, and nomenclature. Although both platforms have contributed to the understanding of sepsis-associated haemostatic abnormalities, the present review specifically focuses on ROTEM. Its multi-assay configuration, including EXTEM, INTEM, FIBTEM, and APTEM, allows the functional contribution of different components of haemostasis to be explored in parallel and therefore provides a particularly useful framework for phenotype-based interpretation in sepsis.

Rotational thromboelastometry provides a dynamic assessment of whole-blood coagulation, following clot formation from its initiation to maximal firmness and subsequent lysis. In routine practice, ROTEM can be performed through different assays, the most used being EXTEM, INTEM, FIBTEM, and APTEM. These assays differ according to the pathway activated or the component of haemostasis selectively inhibited, thereby allowing complementary assessment of clot initiation, propagation, firmness, and fibrinolysis. Unlike conventional coagulation tests, which examine selected components of haemostasis at predefined time points, ROTEM integrates the contribution of plasma coagulation factors, fibrinogen, platelets, and fibrinolytic activity into a continuous viscoelastic profile [9,15,44]. In sepsis, this global approach is particularly relevant because apparently discordant abnormalities may coexist within the same patient, making interpretation of the overall pattern more informative than any single parameter.

### 4.1. EXTEM, INTEM, FIBTEM, and APTEM: What Does Each Assay Tell Us?

EXTEM evaluates coagulation following tissue factor activation and provides a rapid overview of clot initiation, propagation, firmness, and lysis. It is therefore particularly useful for characterizing the overall haemostatic phenotype and has been extensively investigated in septic patients [5,10,11,12,45,46].

INTEM activates the contact pathway and assesses coagulation through the intrinsic system. Although EXTEM and INTEM frequently show concordant abnormalities, their combined interpretation may help distinguish generalized alterations of clot formation from pathway-specific abnormalities and may be particularly informative when clot initiation is prolonged.

FIBTEM is based on EXTEM activation while pharmacologically suppressing platelet contribution to clot formation. The resulting tracing predominantly reflects the fibrin-based component of clot firmness. FIBTEM is therefore useful for determining whether increased or reduced overall clot strength is mainly related to fibrinogen/fibrin contribution rather than platelet-mediated clot strength [21,38].

APTEM represents an EXTEM-based assay performed in the presence of an antifibrinolytic agent. Compared with EXTEM, APTEM better suggests the excessive fibrinolytic activity via improvement or preservation of clot firmness and lysis parameters [47,48]. Conversely, APTEM should not be regarded as a routine marker of the intrinsic pathway or as an independent measure of clot strength [41,49].

### 4.2. Clinically Relevant ROTEM Parameters

The clotting time (CT) represents the interval between test initiation and the achievement of an initial predefined clot amplitude and therefore mainly reflects the initiation phase of coagulation. Prolongation may indicate delayed thrombin generation or reduced coagulation factor activity, whereas shortened or low-normal values may occur in procoagulant states. Importantly, CT alone does not define the global coagulation phenotype.

The clot formation time (CFT) describes the subsequent development of the clot and provides information on the kinetics of clot propagation. Prolonged CFT may accompany hypocoagulable states, whereas more rapid clot development may occur in hypercoagulable profiles.

Early amplitudes, particularly A5 and A10, quantify clot firmness 5 and 10 min after CT, respectively. Because they provide an early estimate of subsequent clot strength, they are particularly attractive in acute care settings, where rapid haemostatic characterization may be preferable to waiting for completion of the entire tracing.

Maximum clot firmness (MCF) represents the maximal strength achieved by the clot and is one of the most extensively studied ROTEM variables in sepsis. Increased MCF has been described in hypercoagulable septic phenotypes, whereas reduced MCF is more frequently observed in advanced coagulopathy and has been associated with adverse outcomes [10,16,19,20,50].

Finally, lysis indices (LIs) and maximum lysis (ML) provide information on clot breakdown. High residual clot firmness at later time points, reflected by elevated LI values and low ML, indicates reduced fibrinolytic activity and may identify fibrinolysis shutdown, a phenotype increasingly linked to organ dysfunction and mortality in sepsis [22,41,49,51]. The main ROTEM assays and parameters, together with their biological meaning and typical alterations across hypercoagulable and hypocoagulable septic phenotypes, are summarized in Table 1.

### 4.3. Reading ROTEM as a Pattern Rather than as Isolated Values

A major advantage of ROTEM in sepsis lies in integrating abnormalities occurring at different stages of clot formation. A prolonged CT, for example, should not automatically be interpreted as global hypocoagulability if subsequent A10 or MCF values are preserved or increased. Conversely, apparently normal clot initiation may coexist with markedly impaired clot firmness or fibrinolysis. Such combinations explain why mixed hypo-hypercoagulable profiles have been described in septic patients and why classification based on isolated variables may be misleading [11,12].

**Figure 1:**
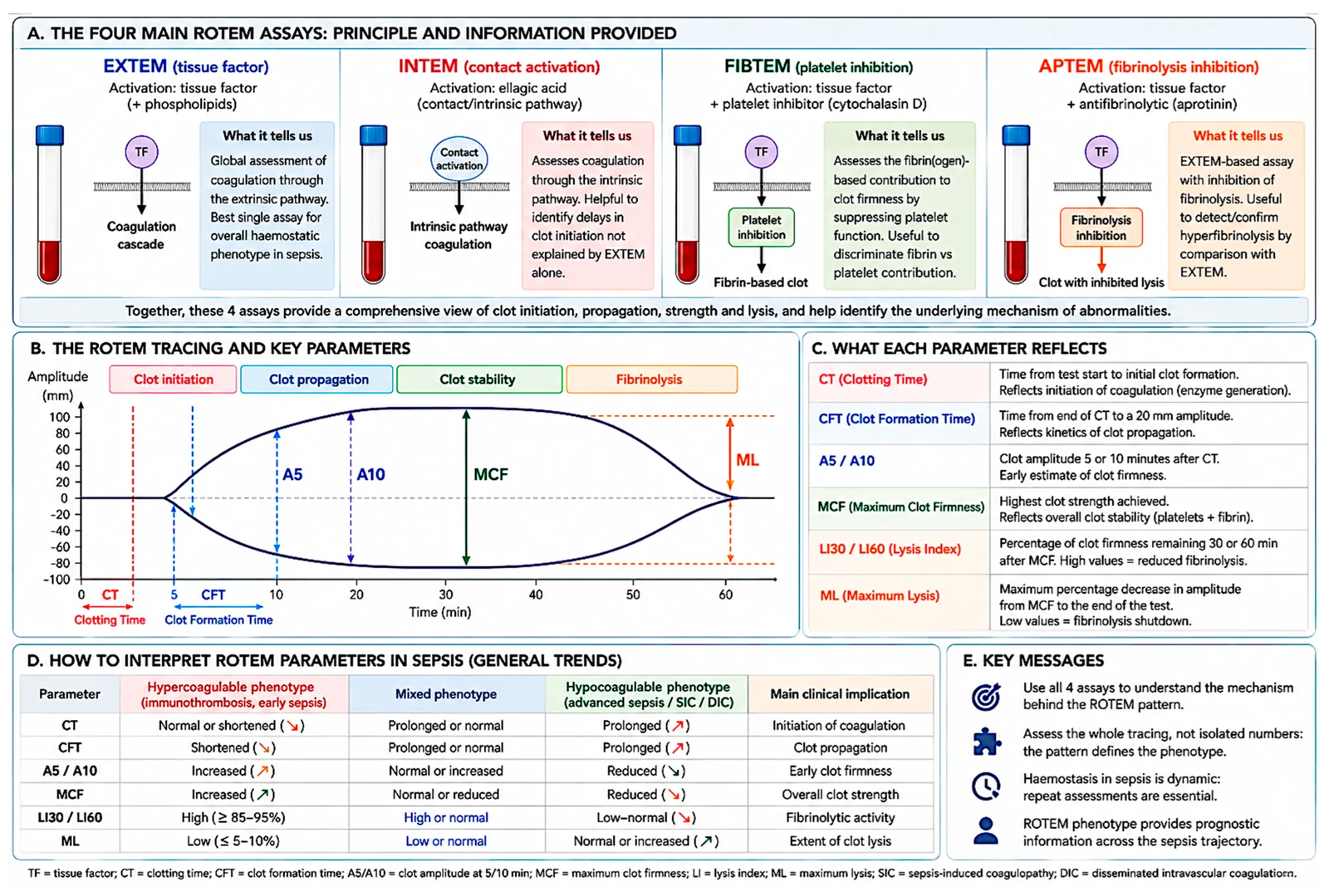
ROTEM assays and key parameters: what they measure and how to interpret them in sepsis.

Accordingly, ROTEM interpretation in sepsis should primarily focus on the overall haemostatic phenotype (clot initiation, propagation, firmness, and lysis) rather than on individual numerical abnormalities. This integrated approach provides the framework for distinguishing the major ROTEM-defined phenotypes encountered across the sepsis spectrum (Figure 1).

## 5. ROTEM-Defined Haemostatic Phenotypes in Sepsis and Septic Shock

ROTEM studies have demonstrated that haemostatic alterations in sepsis cannot be adequately described by a simple distinction between normal and impaired coagulation. Instead, septic patients may exhibit hypercoagulable, hypofibrinolytic, mixed, or hypocoagulable profiles, sometimes despite apparently discordant conventional coagulation tests [5,9,11,12,13,45,46,52]. These patterns should be interpreted as haemostatic phenotypes rather than rigid diagnostic categories, as substantial overlap may occur within the same patient [53,54].

### 5.1. Hypercoagulable Phenotype

**Figure 2:**
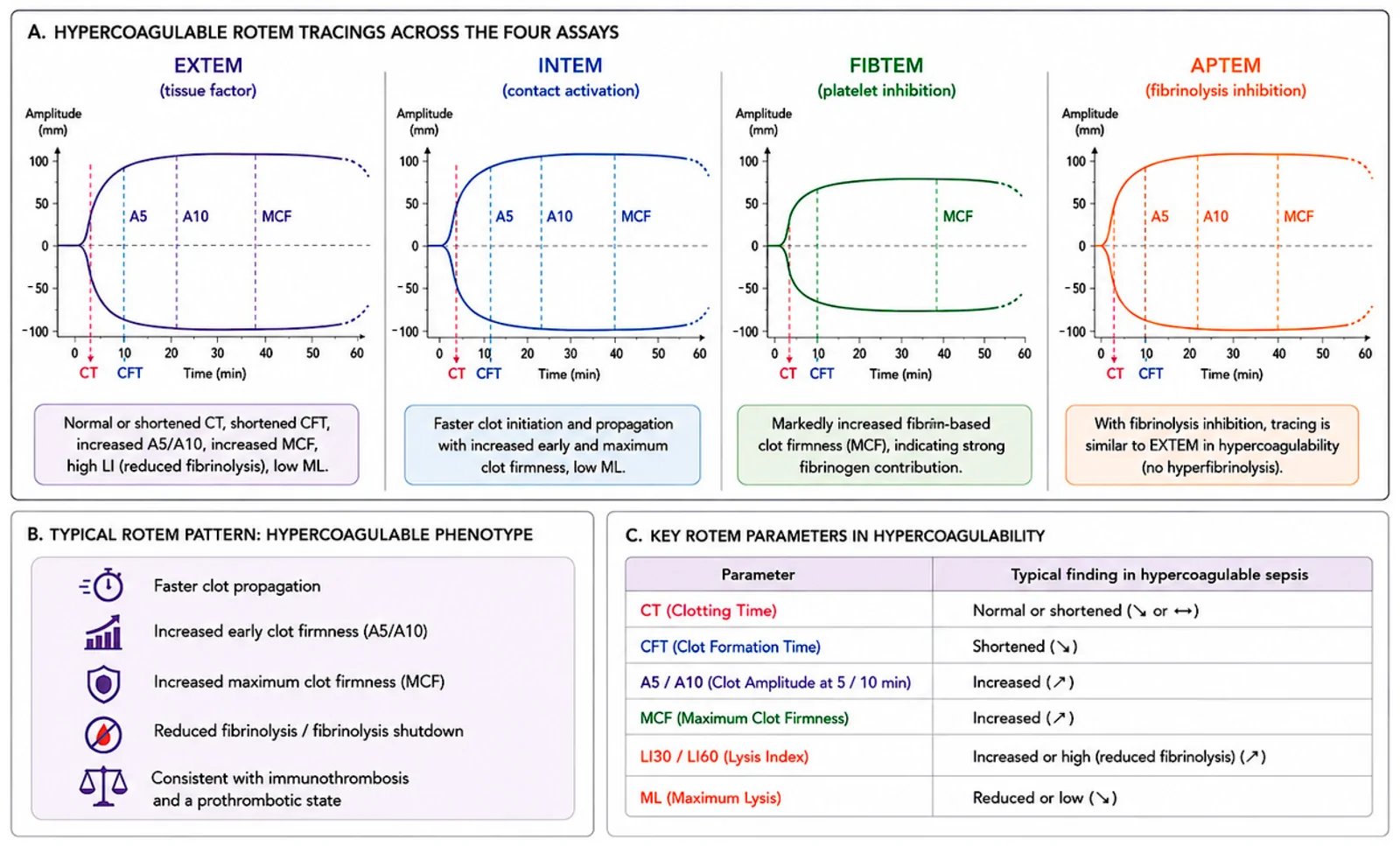
Representative hypercoagulable ROTEM phenotype in sepsis.

Hypercoagulability has been described particularly in early or less advanced sepsis and is predominantly characterized by accelerated clot development and increased clot firmness [45,55,56]. Increased A5/A10 and MCF, especially in EXTEM and FIBTEM, may reflect enhanced fibrin formation and increased clot strength, consistent with the prothrombotic environment generated by immunothrombosis [10,21]. Importantly, hypercoagulability should not be interpreted as preserved or favourable haemostasis. Excessive clot formation may contribute to microvascular thrombosis and impaired tissue perfusion, particularly when associated with suppression of fibrinolysis. A representative ROTEM pattern of septic hypercoagulability is shown in Figure 2.

### 5.2. Fibrinolysis Shutdown

Reduced fibrinolytic activity represents a distinctive and clinically relevant component of septic coagulopathy. On ROTEM, fibrinolysis shutdown is generally reflected by persistent clot firmness over time, high lysis indices, and low ML values [49,51,57,58]. Studies in patients with severe sepsis and septic shock have demonstrated impaired whole-blood fibrinolysis and associations with increasing organ dysfunction and adverse outcomes [22,41,59]. Importantly, fibrinolytic shutdown may coexist with either increased or apparently preserved clot firmness, emphasizing that it represents a partially independent dimension of the haemostatic phenotype rather than simply a feature of hypercoagulability [60,61].

### 5.3. Mixed Hypo-Hypercoagulable Phenotypes

One of the most informative findings provided by ROTEM is the identification of apparently contradictory haemostatic abnormalities within the same patient. A prolonged CT or CFT may coexist with preserved or increased A10 and MCF, indicating delayed clot initiation followed by effective or excessive clot propagation and firmness. In a prospective cohort of patients with sepsis and septic shock, Bui-Thi et al. identified mixed hypo-hypercoagulable patterns that could not be adequately characterized by conventional coagulation testing [12]. Such discordance illustrates why isolated ROTEM variables—or conventional tests alone—may lead to oversimplified classification of septic coagulopathy.

### 5.4. Hypocoagulable Phenotype

**Figure 3:**
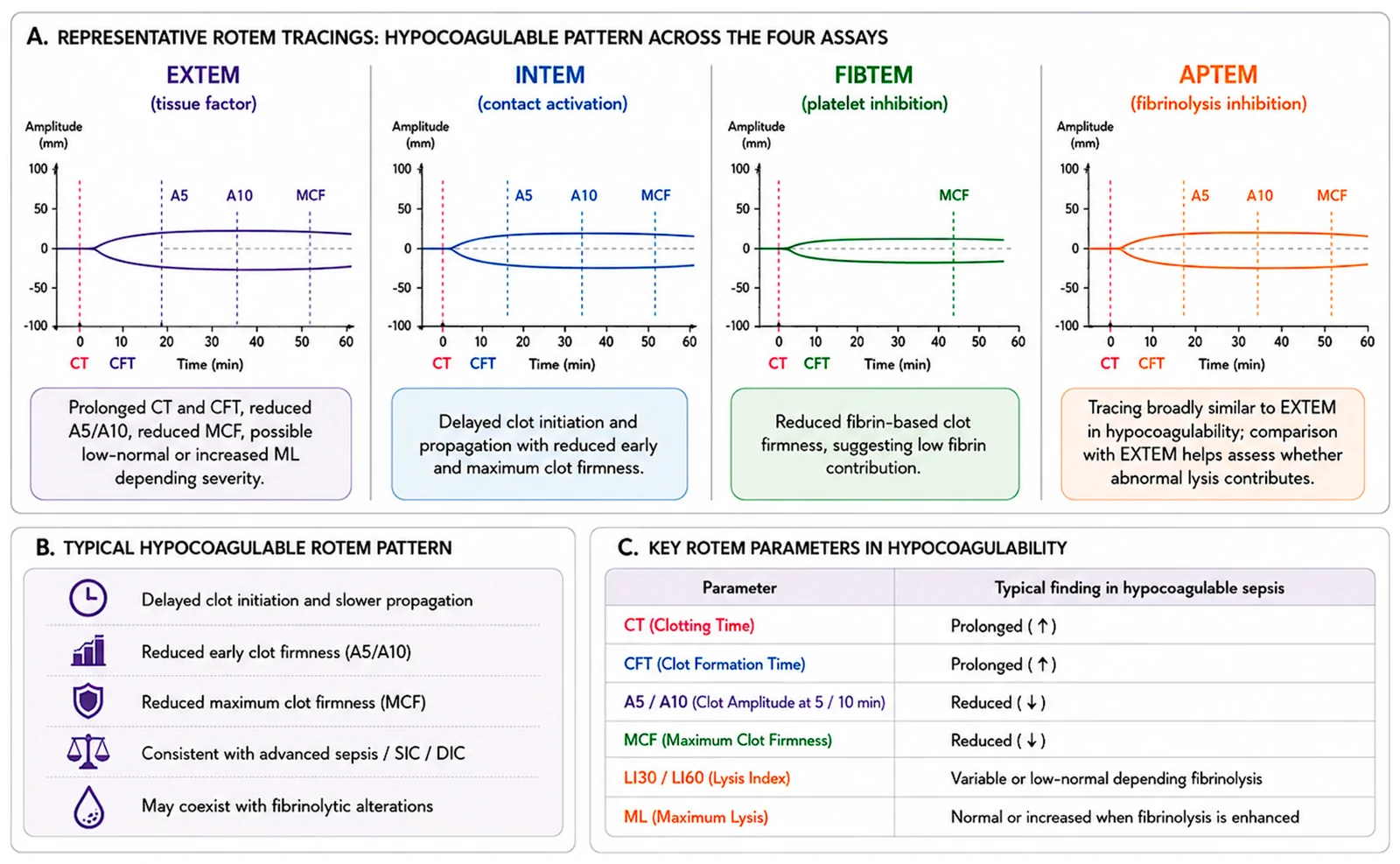
Representative hypocoagulable ROTEM phenotype in sepsis and septic shock.

With increasing severity of sepsis, reduced clot formation and firmness occur more frequently. Hypocoagulable profiles may include prolonged CT and CFT together with decreased A5/A10 and MCF, reflecting impaired thrombin generation, reduced fibrin contribution, platelet dysfunction or consumption, and progressive depletion of coagulation factors. Hypocoagulability appears more frequently in septic shock and advanced coagulopathy than in uncomplicated sepsis [10,11,12,18,26]. Importantly, reduced clot firmness has repeatedly been associated with adverse prognosis, suggesting that transition toward a hypocoagulable phenotype may identify patients with more advanced haemostatic dysfunction [16,17,19,20,62]. A representative hypocoagulable ROTEM phenotype is illustrated in Figure 3.

### 5.5. Consumptive Coagulopathy and Overt DIC

At the most severe end of the spectrum, persistent coagulation activation may lead to consumption of platelets and coagulation factors, reduced clot strength, and overt disseminated intravascular coagulation [26,42,43,63]. ROTEM may demonstrate progressively impaired clot initiation and propagation together with marked reductions in clot firmness, although individual variables can remain within conventional reference ranges even in critically ill septic patients [9,45]. ROTEM should therefore not be considered a substitute for established SIC or DIC diagnostic criteria [64,65,66,67]. Rather, its value lies in providing a functional representation of whole-blood haemostasis that complements conventional laboratory and clinical assessment and may reveal haemostatic abnormalities otherwise undetectable from standard testing alone [68,69,70].

Overall, these observations support a phenotype-based interpretation of septic coagulopathy. Hypercoagulability, fibrinolysis shutdown, mixed patterns, and hypocoagulability should not be viewed as mutually exclusive states, but as potentially overlapping manifestations of a dynamic haemostatic process whose expression may change throughout the course of sepsis.

The clinical evidence supporting these ROTEM- and VHA-defined haemostatic phenotypes is summarized in Table 2. Rather than providing an exhaustive inventory of the literature, the table highlights representative studies illustrating the spectrum from hypercoagulability and mixed patterns to hypocoagulability and impaired fibrinolysis, together with their main associations with disease severity and outcomes.

## 6. From Hypercoagulability to Hypocoagulability: A Dynamic Haemostatic Continuum?

**Figure 4:**
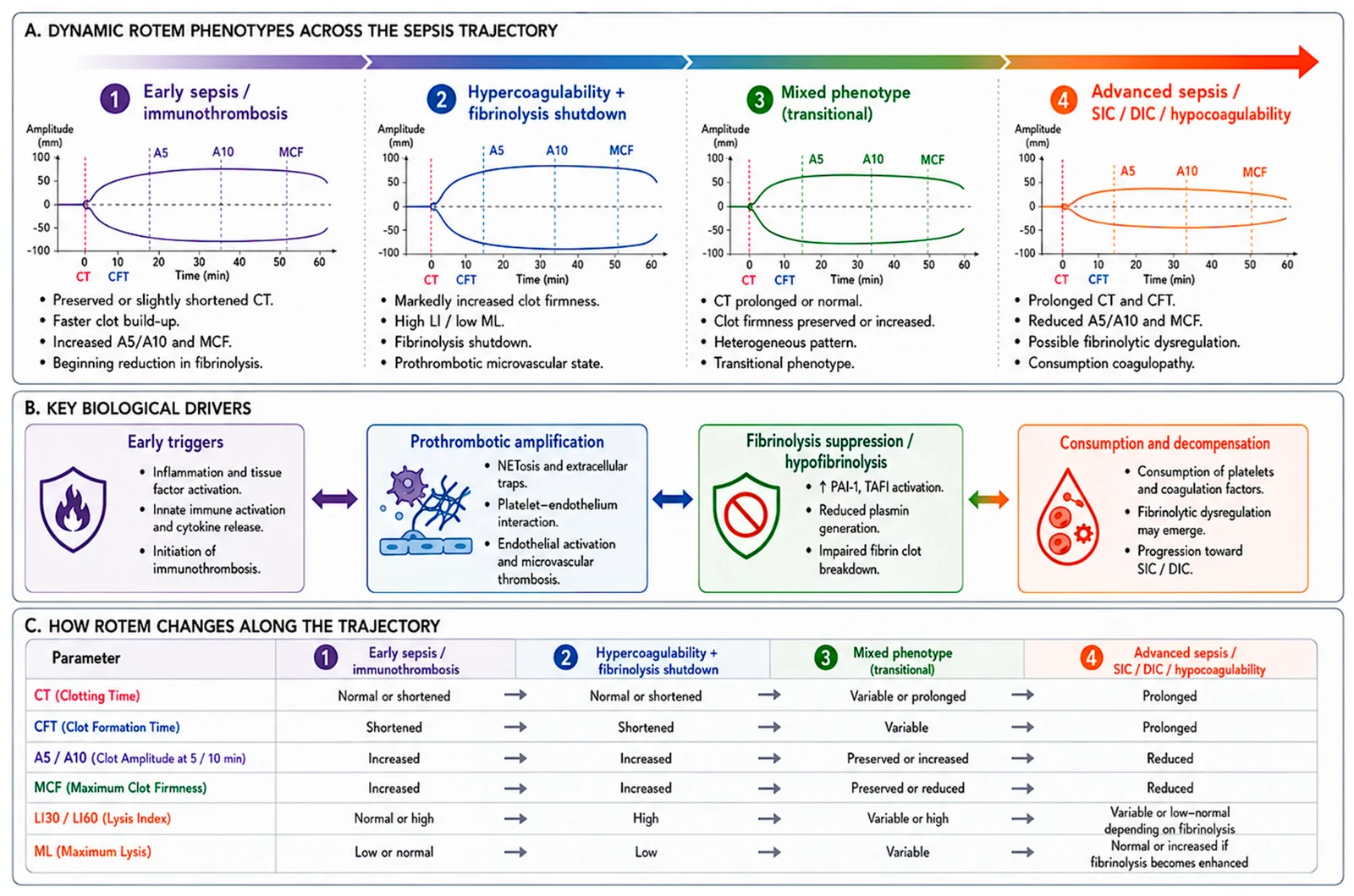
From immunothrombosis to consumptive coagulopathy: a dynamic model of ROTEM phenotypes in sepsis.

The haemostatic abnormalities observed in sepsis are unlikely to represent fixed and mutually exclusive states. Rather, available evidence supports the concept of a dynamic continuum in which the balance between coagulation activation, clot firmness, fibrinolysis, and factor consumption may change during illness [9,10,11,12,17,18]. The proposed relationship among these phenotypes is summarized in Figure 4.

### 6.1. Early Sepsis Versus Septic Shock

Hypercoagulable profiles have been reported more frequently in early sepsis, where increased clot firmness and accelerated clot development may reflect predominant immunothrombotic activation [10,21,45,56]. In contrast, septic shock and more advanced coagulopathy are more commonly associated with delayed clot formation, reduced clot firmness, and hypocoagulable patterns [10,11,12,26,62]. However, this distinction is not absolute. Considerable overlap exists, and patients with severe disease may retain hypercoagulable or mixed features.

### 6.2. Phenotypic Transitions over Time

A biologically plausible trajectory may involve an initial procoagulant and hypofibrinolytic response, followed by progressive consumption of platelets and coagulation factors, impaired clot formation, and eventually overt hypocoagulability or DIC. Importantly, the current evidence does not demonstrate that all septic patients follow this linear sequence. Mixed phenotypes and discordant ROTEM findings suggest that different components of haemostasis may evolve at different rates, producing transitional states rather than discrete stages [11,12,17,18].

### 6.3. Why a Single ROTEM Measurement May Be Insufficient

Most available studies rely on ROTEM assessment at a single time point, thereby providing only a snapshot of an intrinsically dynamic process [9]. A baseline hypercoagulable profile may therefore have a different biological and prognostic meaning depending on whether it resolves, persists, or subsequently evolves toward impaired clot formation and reduced clot strength. Similarly, persistent fibrinolysis shutdown may carry different implications from a transient abnormality. Serial ROTEM assessment could therefore provide information not captured by isolated measurements, potentially allowing identification of haemostatic trajectories associated with disease progression or recovery [17,18]. At present, however, this hypothesis remains insufficiently validated and represents one of the most relevant areas for future prospective investigation.

## 7. ROTEM Phenotypes and Clinical Outcomes

Beyond haemostatic characterization, several studies have investigated whether ROTEM abnormalities are associated with disease severity and clinical outcomes in sepsis. Although available evidence remains heterogeneous and predominantly observational, a consistent signal suggests that impaired clot formation, reduced clot strength, and suppressed fibrinolysis are associated with a worse prognosis [9,16,19,20,71,72,73].

### 7.1. Mortality

Hypocoagulable ROTEM profiles appear to be more frequent among non-survivors [16,17,62]. Reduced clot firmness, particularly lower MCF values, has been associated with increased short-term mortality, while prolonged clot initiation and formation have also been reported in patients with poorer outcomes [11,12,19,20,28]. A systematic review and meta-analysis further supported an association between hypocoagulability assessed by viscoelastic testing and mortality in septic patients [74]. These findings suggest that loss of clot strength may identify a more advanced stage of haemostatic dysfunction.

### 7.2. Fibrinolysis and Outcome

Impaired fibrinolysis may carry independent prognostic significance. Reduced clot breakdown has been associated with increasing organ dysfunction and mortality in severe sepsis and septic shock [41,51,58,59]. More recently, elevated lysis indices and evidence of hypofibrinolysis have been associated with worse outcomes in septic DIC [22]. Derived ROTEM parameters assessing clot growth and fibrinolytic resistance, including t-AUCi, have also shown potential for mortality discrimination, although their clinical applicability remains to be established [49,50].

### 7.3. Organ Dysfunction and Disease Severity

ROTEM abnormalities have also been linked to the severity of organ dysfunction [16,18,27,55]. Progressive hypocoagulability and impaired fibrinolysis have been associated with higher severity scores and more advanced septic coagulopathy, while alterations in platelet and fibrin contributions to clot firmness may parallel worsening systemic illness [39,40,41]. These associations reinforce the concept that haemostatic failure may represent not only an epiphenomenon of severe sepsis, but also a marker of the underlying progression of endothelial and microvascular dysfunction.

### 7.4. Is the Trajectory More Informative than the Baseline Phenotype

Despite these findings, most available studies evaluate ROTEM at a single time point. Consequently, it remains unclear whether prognosis is determined primarily by the baseline phenotype or by its persistence and evolution over time. A patient who remains persistently hypocoagulable or hypofibrinolytic may have a substantially different risk profile from one whose abnormalities rapidly normalize. Whether serial ROTEM trajectories improve prognostic stratification beyond conventional severity scores and isolated measurements remains an important unanswered question and a key target for future prospective studies.

### 7.5. From Prognostic Association to Therapeutic Guidance: An Unmet Evidence Gap

Despite the association between ROTEM-defined phenotypes and clinical outcomes, evidence that viscoelastic phenotyping can improve therapeutic decision-making in sepsis remains lacking. Previous studies have primarily established diagnostic or prognostic associations, without demonstrating that ROTEM-guided interventions translate into improved patient outcomes [9,13]. This distinction is particularly relevant for anticoagulant strategies. Agents targeting sepsis-associated coagulation abnormalities, including antithrombin and recombinant thrombomodulin, appear to provide greater potential benefit in selected patients with sepsis-associated DIC rather than in unselected septic populations [25]. Thus, accurate haemostatic phenotyping may represent a prerequisite for future individualized treatment strategies, but no prospective study has yet demonstrated that ROTEM-guided therapeutic intervention improves outcomes.

## 8. ROTEM Versus Conventional Coagulation Testing

Conventional coagulation tests remain essential in the assessment of septic patients, but they provide only a partial representation of haemostasis. Prothrombin time, activated partial thromboplastin time, platelet count, and fibrinogen primarily describe selected components of coagulation and do not directly assess the kinetics of whole-blood clot formation, clot strength, or fibrinolysis. This limitation is particularly relevant in sepsis, where different components of haemostasis may be altered simultaneously and in opposing directions [5,9,13,75].

### 8.1. What Conventional Tests Measure and What They Miss

Prolonged PT or aPTT may suggest impaired coagulation factor activity, while thrombocytopenia and reduced fibrinogen may indicate consumption and advanced coagulopathy. However, these abnormalities do not necessarily translate into reduced whole-blood clot strength. Conversely, apparently preserved conventional tests may coexist with impaired clot firmness or suppressed fibrinolysis. ROTEM complements conventional testing by integrating clot initiation, propagation, firmness, and lysis into a single functional profile [5,11,12,75,76].

### 8.2. Discordant Coagulation Phenotypes

Discordance between conventional coagulation tests and ROTEM represents one of the most clinically relevant findings in sepsis. Patients classified as hypocoagulable according to standard laboratory abnormalities may still demonstrate preserved or increased clot firmness on ROTEM, revealing a hypercoagulable or mixed functional phenotype [11,12,75,76,77,78]. Such findings highlight the limitations of interpreting septic coagulopathy solely through isolated plasma-based measurements and reinforce the importance of assessing the global haemostatic response.

### 8.3. Complementary Rather than Competing Tools

ROTEM should not be considered a replacement for conventional coagulation testing, but rather a complementary method that provides functional information unavailable from standard laboratory assays. Conventional tests remain important for diagnosis and established SIC/DIC scoring systems [79], while ROTEM may add phenotypic information regarding clot kinetics, firmness, and fibrinolysis. Their combined interpretation may therefore provide a more complete characterization of haemostatic dysfunction in sepsis than either approach alone [5,13].

## 9. Potential Clinical Implications of ROTEM Phenotyping

The identification of distinct ROTEM-defined haemostatic phenotypes may have relevant clinical implications, although these currently remain largely hypothesis-generating rather than treatment-directing.

### 9.1. Bleeding and Transfusion Management

In patients with active bleeding or advanced coagulopathy, ROTEM may help identify whether impaired clot formation is predominantly related to reduced fibrin-based clot strength, platelet contribution, or delayed clot initiation. This information may complement conventional laboratory testing and potentially support more targeted haemostatic replacement [80,81,82]. In this setting, ROTEM may therefore complement conventional testing by identifying the predominant functional defect and supporting a more targeted haemostatic assessment [9,15,74,83].

### 9.2. Anticoagulation and Hypercoagulable Phenotypes

The identification of hypercoagulability or fibrinolysis shutdown does not currently justify escalation of anticoagulant therapy outside established indications. Nevertheless, the observation that anticoagulant strategies such as antithrombin or recombinant thrombomodulin may provide greater benefit in selected patients with sepsis-associated DIC than in unselected septic populations supports the broader concept that haemostatic heterogeneity may influence treatment response [23,84,85]. ROTEM could potentially contribute to such phenotypic stratification, although this hypothesis remains untested in interventional trials [15,86].

## 10. Limitations of the Current Evidence

The current evidence on ROTEM in sepsis is limited by substantial heterogeneity in study populations, sepsis definitions, timing of measurements, devices, reagents, and reference ranges [87]. Most available studies are observational and relatively small, while TEG and ROTEM data are sometimes pooled despite methodological differences [5,9,10,11,12,19,20,45].

Definitions of hypercoagulability, hypocoagulability, and fibrinolysis shutdown also vary considerably across studies, limiting direct comparison and external validity. In addition, most cohorts rely on single-point assessments, whereas sepsis-associated coagulopathy is inherently dynamic. Finally, the scarcity of interventional studies prevents determination of whether specific ROTEM phenotypes are modifiable therapeutic targets or primarily markers of underlying disease severity [9,15,74].

## 11. Future Perspectives: Toward Serial Haemostatic Phenotyping

Future research should move beyond isolated ROTEM measurements toward standardized, serial assessment of haemostatic phenotypes. Prospective studies are needed to determine whether persistent hypercoagulability, fibrinolysis shutdown, mixed phenotypes, or progression toward hypocoagulability identify distinct biological and prognostic trajectories [17,18].

Standardized phenotype definitions and harmonized timing of ROTEM assessment will be essential to improve comparability across studies. Integration of ROTEM findings with clinical severity scores and established SIC/DIC criteria may also clarify whether viscoelastic phenotyping provides incremental prognostic value. Ultimately, prospective interventional studies will be required to determine whether ROTEM-defined haemostatic trajectories can identify treatment-responsive subgroups and translate dynamic phenotyping into clinically meaningful individualized management.

Future prospective studies should also aim to define reproducible ROTEM parameter ranges and phenotype thresholds across different stages of sepsis severity, including sepsis, septic shock, and sepsis-associated DIC. Such studies should evaluate whether combinations of clot initiation, firmness, and fibrinolytic parameters can provide clinically meaningful stratification beyond conventional severity scores and standard coagulation testing.

## 12. Conclusions

Sepsis is characterized by complex, dynamically linked haemostatic responses that cannot be adequately understood by conventional coagulation tests alone. ROTEM can be a tool providing a functional assessment of the wide array of abnormalities spanning from clot initiation, propagation, firmness, and lysis, hence leading to the identification of hypercoagulable, hypofibrinolytic, mixed, and hypocoagulable phenotypes across the sepsis spectrum [9,13,15].

Available evidence suggests that these phenotypes are clinically relevant, with impaired clot strength and fibrinolysis being increasingly associated with disease severity and adverse outcomes [22,41,50,74]. However, ROTEM should currently be regarded primarily as a complementary tool for haemostatic phenotyping and risk stratification rather than as a validated guide to treatment.

Future prospective studies should determine whether serial ROTEM-defined trajectories provide incremental prognostic information, identify biologically and therapeutically distinct subgroups, and ultimately support more individualized management of sepsis and septic shock. Prospective studies are now needed to establish standardized ROTEM ranges and phenotype-specific thresholds associated with different degrees of sepsis severity and clinical risk.

## Figures and Tables

**Table 1:** Clinical interpretation of ROTEM assays and parameters in sepsis.

ROTEM Assay/Parameter	What It Reflects	Typical Hypercoagulable Finding	Typical Hypocoagulable Finding	Potential Clinical Significance
EXTEM	Tissue factor-activated whole-blood coagulation	Faster clot development, ↑ A5/A10, ↑ MCF	Prolonged CT/CFT, ↓ A5/A10, ↓ MCF	Global haemostatic phenotype
INTEM	Contact/intrinsic pathway activation	Accelerated propagation, ↑ clot firmness	Prolonged CT/CFT, ↓ clot firmness	Complements EXTEM; helps characterize delayed clot initiation
FIBTEM	Fibrin-based clot firmness with platelet inhibition	↑ A5/A10/MCF	↓ A5/A10/MCF	Estimates fibrin-based contribution to clot strength
APTEM	EXTEM-based assay with fibrinolysis inhibition	Usually like EXTEM if no hyperfibrinolysis	Improvement vs. EXTEM if hyperfibrinolysis is present	Supports identification of excessive fibrinolysis
CT	Clot initiation	Shortened or low-normal	Prolonged	Reflects initiation/thrombin generation phase
CFT	Clot propagation	Shortened	Prolonged	Reflects kinetics of clot build-up
A5/A10	Early clot firmness	Increased	Reduced	Rapid estimate of subsequent clot strength
MCF	Maximum clot strength	Increased	Reduced	Key marker of hyper-/hypocoagulable phenotype
LI30/LI60	Residual clot firmness during lysis	Increased in hypofibrinolysis	Variable	High values suggest fibrinolysis shutdown
ML	Maximum clot breakdown	Low in fibrinolysis shutdown	Variable or increased if hyperfibrinolysis	Quantifies overall fibrinolytic activity

Note: A5: clot amplitude at 5 min; A10: clot amplitude at 10 min; APTEM: aprotinin-based extrinsically activated ROTEM assay; CFT: clot formation time; CT: clotting time; EXTEM: extrinsically activated ROTEM assay; FIBTEM: fibrin-based extrinsically activated ROTEM assay with platelet inhibition; INTEM: intrinsically activated ROTEM assay; LI30: lysis index at 30 min; LI60: lysis index at 60 min; MCF: maximum clot firmness; ML: maximum lysis; ROTEM: rotational thromboelastometry.

**Table 2:** Representative studies evaluating viscoelastic haemostatic phenotypes and outcomes in sepsis and septic shock.

Study	Design and Population	VHA Platform/Timing	Main Haemostatic Phenotype or Finding	Main Clinical Association
Adamzik et al., 2011 [16]	Prospective observational study in patients with severe sepsis.	ROTEM	Altered whole-blood coagulation profiles with differences between survivors and non-survivors.	ROTEM variables showed prognostic discrimination and were associated with disease severity and survival.
Ostrowski et al., 2013 [17]	Prospective study; 50 patients with severe sepsis.	TEG, serial measurements	Hypocoagulable, normocoagulable, and hypercoagulable profiles were all observed at admission; progressive reduction in clot strength occurred in non-survivors.	Serial deterioration toward hypocoagulability was associated with increased 28-day mortality.
Haase et al., 2015 [18]	Multicenter prospective cohort; 260 patients with severe sepsis.	TEG, repeated for up to 5 days	Progressive hypocoagulability involving several TEG variables.	Hypocoagulability was consistently associated with increased mortality; reduced fibrinogen contribution was also associated with bleeding.
Davies et al., 2018 [10]	Prospective observational study; 100 patients across sepsis, severe sepsis, and septic shock.	ROTEM	Increased clot firmness in sepsis/severe sepsis; prolonged clot development and impaired fibrinolysis in septic shock.	Impaired fibrinolysis in septic shock was associated with 28-day mortality.
Boscolo et al., 2020 [19]	Prospective observational study in severe sepsis.	ROTEM	Whole-blood hypocoagulable profile.	Hypocoagulability was associated with a greater risk of 28-day mortality.
Bui-Thi et al., 2023 [11,12]	Prospective observational study; 161 patients with sepsis or septic shock.	ROTEM within 24 h	Heterogeneous profiles including hypercoagulability, hypocoagulability, hyperfibrinolysis, and mixed hypo-hypercoagulable states.	Demonstrated marked phenotypic heterogeneity and discordance between conventional coagulation tests and whole-blood viscoelastic assessment.
Scarlatescu et al., 2024 [49]	Prospective observational study in septic patients and healthy controls.	Modified ROTEM EXTEM/NATEM with fibrinolytic assessment	Increased resistance to fibrinolysis; prolonged t-AUCi.	t-AUCi identified reduced fibrinolytic activity and may provide a more sensitive assessment of fibrinolysis resistance than conventional lysis indices.
Czempik et al., 2024 [21]	Prospective cohort of patients with early sepsis.	ROTEM	Early alterations in clot initiation, firmness, and fibrinolytic profile.	Selected ROTEM parameters were associated with short-term mortality and supported early haemostatic phenotyping.
Koami et al., 2025 [22]	Retrospective study of septic patients with available ROTEM data.	EXTEM LI60	Hypofibrinolysis/fibrinolysis shutdown.	Hypofibrinolysis was associated with septic DIC, organ dysfunction, and adverse clinical outcomes.

Abbreviations: DIC: disseminated intravascular coagulation; EXTEM: extrinsically activated rotational thromboelastometry; LI60: lysis index at 60 min; NATEM: non-activated rotational thromboelastometry; ROTEM: rotational thromboelastometry; TEG: thromboelastography; t-AUCi: time to attain maximal clot amplitude after reaching maximal clot formation velocity; VHA: viscoelastic haemostatic assay.

## Data Availability

No new data were created or analyzed in this study. Data sharing is not applicable to this article.

## Abbreviations

A5: Clot amplitude at 5 min; A10: Clot amplitude at 10 min; APTEM: Aprotinin-based extrinsically activated ROTEM assay; aPTT: Activated partial thromboplastin time; CFT: Clot formation time; CT: Clotting time; DIC: Disseminated intravascular coagulation; ED: Emergency Department; EXTEM: Extrinsically activated ROTEM assay; FIBTEM: Fibrin-based extrinsically activated ROTEM assay with platelet inhibition; INTEM: Intrinsically activated ROTEM assay; LI: Lysis index; LI30: Lysis index at 30 min; LI60: Lysis index at 60 min; MCF: Maximum clot firmness; ML: Maximum lysis; PT: Prothrombin time; ROTEM: Rotational thromboelastometry; SIC: Sepsis-induced coagulopathy; SOFA: Sequential Organ Failure Assessment; TEG: Thromboelastography; t-AUCi: Time to attain maximal clot amplitude after reaching maximal clot formation velocity; VHA: Viscoelastic haemostatic assay.
